# Pulmonary Consumption Successfully Treated with Naphtha

**Published:** 1843-11-11

**Authors:** 


*Pulmonary Consumption Successfully Treated with Naptha.* By John Hastings, M. D., Senior Physician to the Blenheim Street Free Dispensary. London: 1843. 8vo. pp. 120.

This pamphlet is the most unblushing piece of effrontery that it has been our lot, for some time, to come across. As the name of Hastings is authority in this country, and as a condensed summary of the work has been extensively circulated by a well known druggist's firm, of a neighbouring city, we deem it our duty to warn our readers of imposition and fraud, and to state to them the real character of the book. In the first place, the work is *not* by Dr. Charles Hastings, of Worcester, the author of the Treatise on Inflammation, and who is so well know throughout these States, but by some most impertinent charlatan, connected with a London Free Dispensary.

From the first moment Dr. Hastings prescribed naphtha in phthisis, he became eonvinced that it would turn out to be little less than a specific in the earlier stages of the disease, and seems equally sanguine, (upon grounds he does not consider himself justified in stating), that, even in the latter stages of the disease, a restoration to health may generally be calculated on. To sustain these formidable assertions from an individual so perfectly

"To fortune and to fame unknown,"

facts are required, and we, accordingly, have detailed, thirty-seven cases, all of which presented (according to the reporter) both rational and physical signs of the most unequivocal import. Along with great emaciation, copious perspirations, profuse expectoration, cough, dyspnœa, there was partial and general dulness over the superior portions of the chest, with characteristic alteration, or absence of the respiratory murmur. Now, of these thirty-seven cases, a perfect cure was effected in thirty-six, from the thirtieth to the eightieth day, (in one case only was the cure protracted to the one hundred and tenth day), and this too by the use of *naphtha*, given in doses of from ten to forty drops three times a day. The results in the thiry-seventh case are thus stated, and are a good specimen of the style of the entire work.

"After a lapse of sixteen days the cough, expectoration, and difficulty of breathing had diminished, the night sweats and other signs of hectic fever had disappeared for rather more than a week, the appetite was natural, and a dry blowing respiration was well marked over the space occupied by the cavern. The dose of naphtha was now increased to twenty drops. On the twentieth day he had a return of all his former symptoms, owing to his getting wet and allowing his clothes to dry on him. The cavern, nevertheless, below the cavicle, could hardly be detected. * * * By the thirty-fourth day a further amendment had evidently ensued, for percussion yielded a much clearer sound, and the respiratory murmur was improved. The cavernous râle was less distinct, and pectoriloquy, could not be detected. Percussion elicited a good sound throughout the chest." p. 89.

Our feelings, after the perusal of this work of matchless bronze, were more those of pity and contempt than of indignation. Our readers will, we feel assured, sustain us in rejecting, as utterly worthless, all the testimony of Dr. Hastings on the efficaey, real or supposed, of naphtha in the treatment of consumption. It may be, for ought we know, a certain specific in phthisis, inchoative, or senile; but Dr. Hastings has signally failed in giving us one tittle of evidence in its favour, and his reprehensible conduct, serves to cast a bad odour on an article which may, eventually, prove useful amongst the Materia Medica.

